# Saffron Extract-Induced Improvement of Depressive-Like Behavior in Mice Is Associated with Modulation of Monoaminergic Neurotransmission

**DOI:** 10.3390/nu13030904

**Published:** 2021-03-11

**Authors:** Camille Monchaux De Oliveira, Line Pourtau, Sylvie Vancassel, Camille Pouchieu, Lucile Capuron, David Gaudout, Nathalie Castanon

**Affiliations:** 1 INRAE, Nutrition and Integrative Neurobiology (NutriNeuro), UMR 1286, 33076 Bordeaux, France; c.monchaux@activinside.com (C.M.D.O.); sylvie.vancassel@inrae.fr (S.V.); lucile.capuron@inrae.fr (L.C.); 2 Department of Life Science and Health, Nutrition and Integrative Neurobiology (NutriNeuro), Bordeaux University, UMR 1286, 33076 Bordeaux, France; 3 Activ’Inside, 33750 Beychac-et-Caillau, France; l.pourtau@activinside.com (L.P.); c.pouchieu@activinside.com (C.P.); d.gaudout@activinside.com (D.G.)

**Keywords:** mood disorders, nutritional interventions, saffron extract, Safr’Inside^TM^, depressive-like behavior, monoaminergic neurotransmission

## Abstract

Depressive disorders represent a major public health concern and display a continuously rising prevalence. Importantly, a large proportion of patients develops aversive side effects and/or does not respond properly to conventional antidepressants. These issues highlight the need to identify further therapeutic strategies, including nutritional approaches using natural plant extracts with known beneficial impacts on health. In that context, growing evidence suggests that saffron could be a particularly promising candidate. This preclinical study aimed therefore to test its antidepressant-like properties in mice and to decipher the underlying mechanisms by focusing on monoaminergic neurotransmission, due to its strong implication in mood disorders. For this purpose, the behavioral and neurobiochemical impact of a saffron extract, Safr’Inside™ (6.5 mg/kg *per os*) was measured in naïve mice. Saffron extract reduced depressive-like behavior in the forced swim test. This behavioral improvement was associated with neurobiological modifications, particularly changes in serotonergic and dopaminergic neurotransmission, suggesting that Safr’Inside™ may share common targets with conventional pharmacological antidepressants. This study provides useful information on the therapeutic relevance of nutritional interventions with saffron extracts to improve management of mood disorders.

## 1. Introduction

Mood disorders are one of the most prevalent psychiatric disorders and currently represent a leading cause of disability worldwide [1]. They include major depression, but also subclinical depression as low mood, which is characterized by less severe and/or lasting symptoms than major depression, while being still highly debilitating. To worsen the picture, the remission rate remains particularly low in a significant proportion of depressed patients, despite the large panel of antidepressant (ADs) medications currently available [2,3]. In addition, important side effects that are directly linked to these pharmacological treatments often occur, limiting in turn the increasing dosage regimen classically recommended to optimize therapeutic response [4,5]. These issues promote heightened interest in identifying new strategies relevant to manage depressive symptoms in the largest proportion of patients, while reducing side effects. 

For this purpose, nutritional interventions based on the use of essential nutrients or bioactive natural ingredients, extracted from plants with known beneficial effects on health and mood, may be an interesting alternative to chemical drugs [6,7]. In that context, mounting evidence points to saffron as a particularly promising candidate [8,9]. Saffron is a spice derived from the dried stigmas of flower of *Crocus sativus* L. This historic spice, renowned for its culinary properties, has also been used for centuries for its positive impact on health [10]. Indeed, it contains a large number of biologically active compounds, the four main ones being crocin and crocetin, two carotenoid pigments responsible for its color; picrocrocin, which provides its flavor and bitter taste; and safranal, a volatile compound responsible for its aroma and smell. Thus, these main constituents likely contribute not only to the sensory profile of saffron but also to its therapeutic properties [10], as shown in the treatment of various medical conditions, such as arthritis, menstrual disorders, liver disease, or dental pain [11]. Different saffron compounds have also been shown to display antioxidant, anti-inflammatory and, importantly, anxiolytic and/or antidepressant properties [12,13,14,15]. Several clinical studies have shown that administrating saffron extracts alone [8,9] or combined with classical ADs [16] improves mood in patients suffering from mild to moderate depression. Moreover, a meta-analysis reported that chronic saffron administration (30 mg/day for 6 weeks) to patients with major depressive disorders ameliorates their symptomatology as compared to placebo and can be even as efficient as different conventional ADs (fluoxetine, imipramine) [17]. Supporting further these findings, some preclinical studies described a reduction in depressive-like and anxiety-like behaviors following administration of saffron extracts [18,19] or saffron active compounds [20,21]. Nevertheless, the doses used in these studies were usually very high and not comparable at all with those clinically administered in humans. In addition, the neurobiological mechanisms potentially underlying the reported antidepressant effects of saffron are only very rarely investigated and remain still largely unknown. However, answering this question seems essential to better understand the therapeutic effect of nutritional interventions with saffron.

Based on the current knowledge on the mechanisms of action of standard ADs, it can be hypothesized that saffron could share with these pharmacological treatments some neurobiological targets, including serotonin (5-HT) and/or dopamine (DA) neurotransmission, which play a notorious role in the pathophysiology and treatment of depressive disorders. Conventional pharmacotherapy ultimately aiming to restore brain 5-HT neurotransmission is indeed the first-line treatment to tackle depressive symptoms [22,23], although most serotonergic ADs are also known to improve other pathophysiological mechanisms of depression, such as altered function of the hypothalamo-pituitary-adrenal (HPA) axis or impaired neuroplasticity [24]. More recently, compelling evidence has also pointed to impairment of DA neurotransmission as an additional important contributor to the development of key symptoms of mood disorders [24,25,26,27], especially those related with apathy or reduced motivation [28,29]. In agreement with these findings, it has been shown that some classical ADs (e.g., desipramine or fluoxetine) increase brain DA levels [26], while drugs directly targeting the dopaminergic system (e.g., bupropion) display antidepressant properties [26,30]. All together, these findings highlight the central role of serotonergic and dopaminergic systems in the pharmacological treatment of depressive disorders. Importantly, they argue for the potential implication of these monoaminergic systems in the antidepressant properties of saffron, although its neurobiochemical impact is largely unknown.

In order to address this issue, the present study aimed to measure in mice the effects of both acute and chronic oral administration of Safr’Inside^TM^, a standardized saffron extract, on emotional behaviors as measured in different complementary and well-validated behavioral tests assessing depressive-like and anxiety-like behaviors, which model two major symptomatic dimensions of depression [31]. Importantly, we also aimed to understand the neurobiological mechanisms underlying the expected behavioral improvement by concomitantly measuring its impact on some of the main neurobiological systems known to be targeted by standard ADs, namely serotonergic and dopaminergic systems. We showed that Safr’Inside^TM^ reduces depressive-like behavior. Moreover, we provided interesting evidence pointing to the involvement of monoamine modulations in the reported behavioral improvement.

## 2. Materials and Methods

### 2.1. Animals and Housing Conditions

Eight-weeks old male C57BL/6J mice were obtained from Janvier labs (France). Upon arrival, mice were randomly allocated to control or Safr’Inside groups and single-housed with enrichment in a controlled environment (22 °C ± 2 °C, 40% of humidity), with a 12 h/12 h light/dark cycle (light on at 7:30 a.m.). Mice had ad libitum access to water and food (Standard Rodent Diet A04, SAFE, Augy, France). No body weight differences existed between the two groups at the experiment onset. All experiments were conducted in strict compliance with the European Union recommendations (2010/63/EU) and were approved by the local ethical committee (approval ID A16873). Maximal efforts were made to reduce the suffering and the number of animals used.

### 2.2. Oral Administration of Safr’Inside^TM^

The saffron extract (Safr’Inside^TM^) provided by Activ’Inside company (Beychac-et-Caillau, France) is a Safromotivines^TM^ standardized extract containing more than 25 active compounds, including safranal (>0.2% according to U-HPLC method [32]). Safr’Inside^TM^ or its vehicle (water) for control mice were orally administered in the morning by gavage through a flexible tube kindly inserted into the animal’s digestive system (mouse-adapted feeding probes; ECIMED 1.33 × 30 mm). The volume of injection was 10 mL/Kg, in accordance with previously published recommendations [33]. All solutions were brought to room temperature before administration to avoid decreasing body temperature during this process. Mice were handled and accustomed to the gavage procedure for several days prior to the test. For acute experiments, the solutions were administered 30 min before the behavioral assessment. For the chronic experiment, mice received one gavage per day for 4 weeks, the last being done 3 h before the behavioral test.

### 2.3. Experimental Design

In a first experiment, 30 mice were used to determine an acute effective dose of Safr’Inside^TM^ in the forced swim test (FST). Based on a preliminary study testing three doses (6.25, 150 and 600 mg/Kg; unpublished data), two of them were chosen for the present experiment: 6.25 and 600 mg/Kg. The low one was calculated based on the ratio given by the United States Food and Drug Administration [34] to reflect, for a mouse, the equivalent of the effective dose classically administrated to humans, namely 30 mg/day [15]. The high dose was selected on the basis of previously published data [18]. 

**Figure 1:**
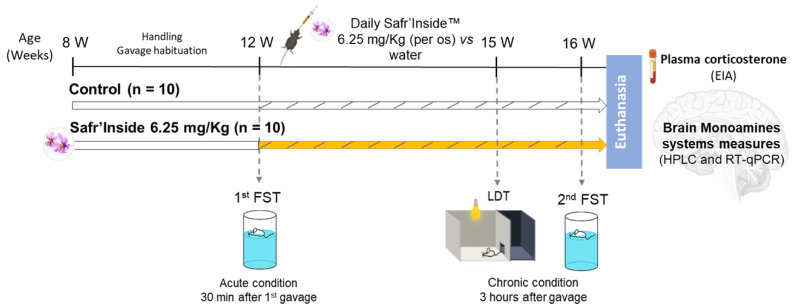
Experimental design to test the effects of acute and chronic administration of Safr’Inside^TM^ (6.25 mg/Kg) on depressive-like behavior and brain monoamines systems.

In a second experiment, 20 naïve mice were used to investigate the effects of acute and chronic administration of Safr’Inside^TM^ on depressive-like and anxiety-like behavior, and brain monoaminergic systems. The dose of 6.25 mg/Kg *per os* was used based on data from the first experiment. Here, mice were tested in the FST a first time 30 min after the first administration of saffron extract or water (Acute condition). Then, after 3 weeks of daily treatment, they were tested in a light–dark test (LDT) and again in the FST 1 week later (Chronic condition) (Figure 1).

### 2.4. Behavioral Tests 

All behavioral procedures were performed during the light cycle, in a dedicated sound-proof behavioral facility as previously described [35,36]. All testing equipment was thoroughly cleaned between each session. Both depressive-like and anxiety-like behaviors were assessed in order to measure the impact of saffron on a large panel of emotional behaviors and to reveal potential behavior-related specificity. When two behavioral tests were carried out in the same mice, we were careful to start with the least stressful of the two (LDT as compared to FST) and to apply a between-test interval of 1 week, in order to avoid potential interferences between tests.

*Depressive-like behavior in the forced swim test (FST).* Depressive-like behavior was assessed in the FST, a standardized and validated rodent test to predict the antidepressant activity of drugs [37]. Briefly, mice were placed individually in a cylinder (diameter: 16 cm; height: 31 cm) half-filled with 25 °C (+/−1 °C) water for a 6-min test. Duration of swimming, climbing and immobility was determined during the last 4 min of the test [38]. A mouse was judged to be immobile when it moved only slowly to remain floating, keeping its head above water. Increased duration of immobility has been proposed to reflect a state of helplessness that is reduced by ADs. Behavior was videotaped to be scored later by a trained observer blind to treatment conditions, using ‘‘The Observer XT 11’’ software (Noldus, Wageningen, The Netherlands). An immobility threshold that represents the average percentage of time spent immobile by control animals was calculated for each experiment. We then determined an immobility index reflecting the percentage of mice spending more or less immobility time than the immobility threshold. 

*Anxiety-like behavior in the light/dark test (LDT).* The LDT is a standardized test to assess anxiety in rodents. As previously described [39], it is based on their innate aversion to brightly lit areas, and their spontaneous exploratory behavior when exposed to a novel environment. In this procedure, an increase in time spent in the light compartment is considered to reflect an anxiolytic activity. The apparatus has two compartments connected by a 7 cm door; a small covered and dark compartment (brightness < 5 lux, one third of the total box) and a large light aversive compartment (brightness ≈ 300 lux, two thirds of the box). Mice were placed in the dark compartment at the beginning of the procedure. The door between the two compartments was opened and the latency to reach the light box, as well as the time spent in each compartment were automatically measured for an 8-min test using a video tracking system (SMART system; Bioseb, Vitrolles, France). The covered distance in the light box was also automatically measured and normalized to the time spent in this compartment in order to assess locomotion.

### 2.5. Tissue Sampling

Immediately after the last FST, mice were anesthetized with an intraperitoneal injection of pentobarbital/lidocaine solution (300 and 30 mg/Kg respectively). Blood samples were collected via cardiac puncture into 10% EDTA-coated tubes. After centrifugation (20 min, 2000× *g*, 4 °C), aliquots of plasma were stored at −80 °C for further analysis. Mice were then transcardially perfused with chilled PBS 1× (2 min, 10 mL/min) to remove all traces of blood from tissues. Brains were rapidly extracted from the skulls and carefully dissected to hemilaterally collect the frontal cortex (FCx), striatum (Str) and hippocampus (HPC). Brain structures were immediately frozen with dry ice and stored at −80 °C.

### 2.6. Enzyme Immunoassays (EIA) for Corticosterone Dosage

Plasma corticosterone levels were assessed using Corticosterone-HS kit (ImmunoDiagnostic System, Pouilly, France) following the manufacturer’s instructions. All sample were diluted 10× with calibrator diluent and run in duplicates. The absorbance was measured at 450 nm by spectrophotometry (Victor3V, PerkinElmer, Villebon-sur-Yvette, France). Corticosterone concentration of each sample was calculated according to the standard range provided by the supplier, and expressed in ng/mL.

### 2.7. Brain Monoamines and Metabolites Analysis by High Performance Liquid Chromatography Coupled to Electrochemical Detection (HPLC-EC)

Half FCx, Str and HPC were homogenized at 4 °C (3 × 1 min at 30 Hz) using a TissuLyser system (Qiagen, Courtaboeuf, France) in 600 µL of extraction buffer (containing 12 mM perchloric acid, 56 µM EDTA, 0.26 mM sodium disulfite and 3 mM octanesulfonic acid, as previously described [40]). Homogenates were centrifuged (20 min, 4 °C, 16,000× *g*) and the supernatant containing the monoamines was recovered, centrifuged on filter tubes (2 min, 4 °C, 1600× *g*) and then stored at −80 °C until use. For the assay, 20 µL of supernatant from each sample were injected into a high-performance liquid chromatograph equipped with a 5 μm C18, 3 × 100 mm silica column (ACE, AIT, Cormeilles-en-Parisis, France) and a DECADE II electrochemical detector (Antec Leyden, Zoeterwoude, The Netherlands). The flow rate of the pump allowing the circulation of the mobile phase (0.1 M sodium acetate, 0.1 M citric acid, 1 mM diethylamine, 1 mM sodium octyl sulfate and 0.1 mM EDTA) was set at 0.3 mL/min. The monoamines of interest, DA, 5-HT, and their metabolites, dihydroxyphenylacetic acid (DOPAC), homovanillic acid (HVA) and 5-hydroxyindoleacetic acid (5-HIAA), were identified according to their retention time, as compared to their respective standards injected daily using the Chromeleon integration 6.8 software (Dionex, Sunnyvale, CA, US). The different standards were mixed in a standard solution, which therefore contained a known concentration of each monoamine (DA-hydrochloride (H8502) and 5-HT-hydrochloride (H9523), Sigma, St. Quentin Fallavier, France) and each metabolite (DOPAC (850217), HVA (H1252) and 5-HIAA (H8876), Sigma, St. Quentin Fallavier, France), allowing the calculation of the contents to assay. Each sample was weighed accurately beforehand and the results were expressed for each sample in pmoles/g of fresh tissue.

### 2.8. Real-Time Quantitative PCR (RT-qPCR) 

The expression of the different genes of interest was evaluated by RT-qPCR. Total RNAs was extracted from half FCx, Str and HPC using Trizol (Invitrogen, Life Technologies, Villebon-sur-Yvette, France) as previously described [41]. The purity and quantity of RNA were measured by spectrophotometry for each sample (Nanodrop, Life technologies, Villebon-sur-Yvette, France). Then, 2 µg of RNA pre-treated with RNasin^®^ Ribonuclease Inhibitor (Promega, Charbonnières-les-Bains, France) was reverse-transcribed into complementary DNA using Superscript III (Invitrogen, Life Technologies, Villebon-sur-Yvette, France). 2 µL of cDNA at 20 µg/µL were used in duplicate for the amplification. Genes amplification were performed using the Taqman LightCycler^®^ 480 Probes Master mix (Roche Diagnostics, Meylan, France) and appropriate FAM-labeled Taqman primers (ThermoFisher Scientific, Waltham, MA, US). We focused on genes coding for 5-HT receptors (5-Htr1a: Mm00434106_s1 and 5-Htr1b: Mm00439377_s1) and the 5-HT transporter (SERT: Mm00439391_m1) for 5-HT pathway, DA receptors (DRD1: Mm01353211_m1 and DRD2: Mm00438545_m1), the enzyme Catechol-O-Methyltransferase (COMT: Mm00514377_m1) and the DA transporter (DAT: Mm00438388_m1) for DA pathway, and the monoamine oxidases (Maoa: Mm00558004_m1 and Maob: Mm00555412_m1) that catabolize both 5-HT and DA. Fluorescence was determined on a Light cycler 480 II system (Roche Diagnostics, Meylan, France). The PCR program consisted of 1 cycle of 95 °C for 10 min, 50 cycles of 95 °C for 10 s and 60 °C for 30 s, and 1 cycle of 40 °C for 30 s. Data were analyzed using the comparative threshold cycle method as previously described [42] and results were expressed as relative fold change with GAPDH as a house-keeping gene (Mm99999915_g1).

### 2.9. Statistical Analysis

All data are presented as means ± SEM. Data were analyzed using Statistica 6 software (StatSoft, Tulsa, OK, USA), and possible outliers were assessed with Graphpad Outlier Calculator [43] and removed from the analysis. Normality was assessed using the Shapiro-Wilk test. When distribution was normal data were analyzed with parametric statistics; unpaired t-test and one-way ANOVA, followed by Fisher LSD post-hoc test when appropriate, for comparison of 3 groups. For non-normal distribution, data were analyzed with non-parametric statistics; statistical validity was assessed with the Mann–Whitney U test for comparison of 2 groups and with the Kruskal–Wallis H test for comparison of 3 groups. The immobility indexes were analyzed using Fisher’s exact test on contingency tables. *p* values ≤ 0.05 denote statistical significance.

## 3. Results

### 3.1. Efficient Dose of Acute Oral Administration of Safr’Inside^TM^ in the FST 

**Figure 2:**
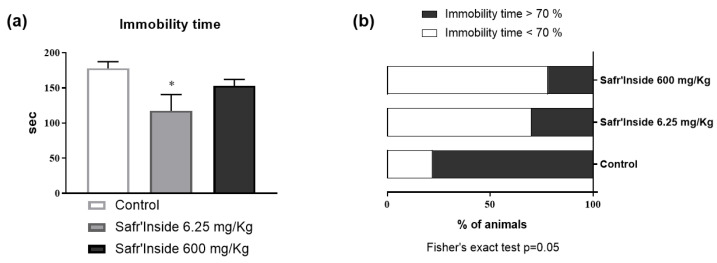
Effect of acute oral administration of Safr’Inside^TM^ (6.25 and 600 mg/Kg) on depressive-like behavior measured in the FST. (**a**) Immobility time; (**b**) Index of immobility. FST has been conducted 30 min after gavage. Results are shown as mean ± SEM. Control (Water): *n* = 9; Safr’Inside (6.25 mg/Kg): *n* = 10; Safr’Inside (600 mg/Kg): *n* = 9. * *p* < 0.05.

In order to define a behaviorally effective dose of Safr’Inside to use in the next experiment, we compared the impact of a low dose (6.25 mg/Kg) and a high dose (600 mg/Kg) on depressive-like behavior, as assessed in the FST, a classical rodent test of depression [37]. Although the main effect of an acute Safr’Inside administration does not reach statistical significance (Figure 2a; *p* = 0.07), it differentially changes immobility time depending on the dose. This effect is especially notable in mice that received the low dose (6.25 mg/Kg), as shown by the group-by-group comparison revealing a significant effect of this dose as compared to controls (*p* < 0.05). We also calculated an immobility index that reflects the percentage of mice spending more than 70% of their time immobile (Figure 2b). The statistical analysis showed that treatment significantly changes this index (*p* ≤ 0.05). As expected, it is particularly elevated in the control group (77.8%) as compared to mice who received the low (30.0%) or the high (22.2%) dose of Safr’Inside. Based on these data, the lowest dose (6.25 mg/Kg *per os*) was chosen for the next experiment, since it does improve depressive-like behavior and appears as most relevant for translational research than the high one.

### 3.2. Impact of Acute and Chronic Oral Administration of Safr’Inside^TM^ on Depressive-Like and Anxiety-Like Behaviors

**Figure 3:**
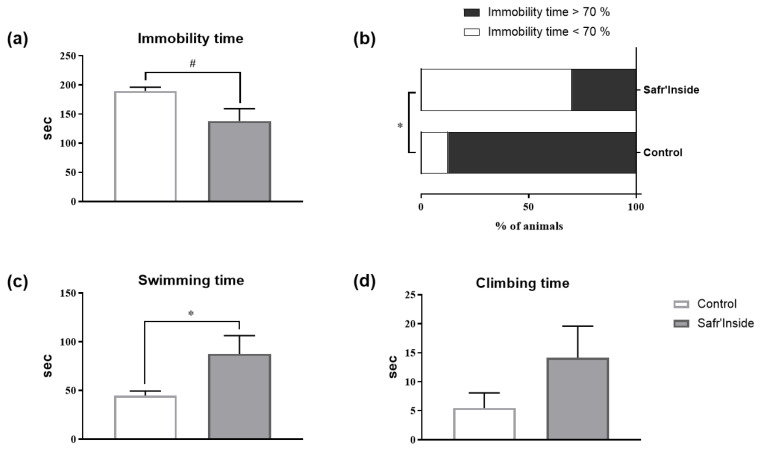
Effect of acute oral administration of Safr’Inside^TM^ (6.25 mg/Kg) on depressive-like behavior measured in the FST. (**a**) Immobility time; (**b**) Index of immobility; (**c**) Swimming time and (**d**) Climbing time. FST has been conducted 30 min after gavage. Results are shown as mean ± SEM. Control (Water): *n* = 8; Safr’Inside: *n* = 10. ^#^ *p* = 0.05, * *p* < 0.05.

**Figure 4:**
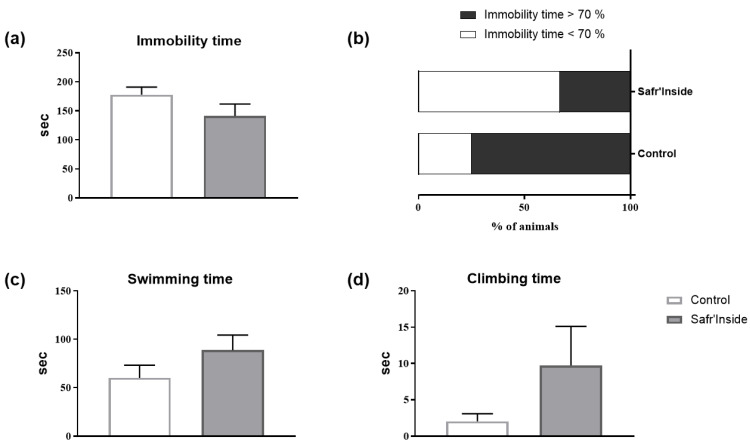
Effect of chronic oral administration of Safr’Inside^TM^ (6.25 mg/Kg) on depressive-like behavior measured in the FST. (**a**) Immobility time; (**b**) Index of immobility; (**c**) Swimming time and (**d**) Climbing time. FST has been conducted after 4 weeks of treatment and 3 h after the last gavage. Results are shown as mean ± SEM. Control (Water): *n* = 8; Safr’Inside: *n* = 9.

We then measured the detailed behavioral (depressive-like and anxiety-like behavior) and neurochemical consequences of chronic Safr’Inside administration (6.25 mg/kg/day), in order to study further its impact on emotional behavior, and to start deciphering the underlying mechanisms. We first checked however the efficiency of the selected dose by testing mice in the FST 30 min after the first administration. Akin to results found in the first experiment (Figure 2), acute Safr’Inside administration significantly reduces immobility time in the FST (Figure 3a; *p* ≤ 0.05). This was confirmed by a significant reduction of the immobility index in the Safr’Inside group as compared to controls (Figure 3b; *p* < 0.05). Conversely, Safr’Inside-treated mice spend more time swimming (Figure 3c; *p* < 0.05), while no significant effect was reported regarding duration of climbing that remains very low in the two groups (Figure 3d; *p* > 0.1). Together, these results support an antidepressant-like effect of acute Safr’Inside administration in this test. Although not reaching statistical significance, similar results were obtained following chronic Safr’Inside treatment, when the same mice were tested again in the FST 5 weeks later (Figure 4).

**Figure 5:**
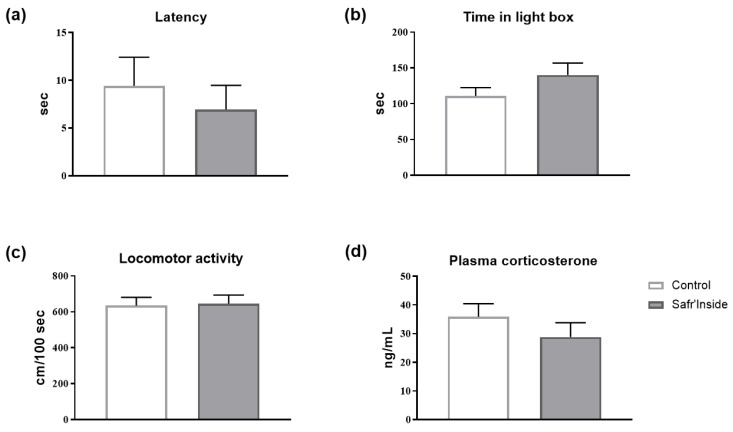
Effect of chronic oral administration of Safr’Inside^TM^ (6.25 mg/Kg) on anxiety-like behavior, measured in the LDT, and plasmatic corticosterone levels. (**a**) Latency to enter in the light box of the LDT; (**b**) Time in light box; (**c**) Locomotor activity as assessed by the distance covered (cm) normalized for the time spent in the light box; (**d**) Plasma corticosterone levels in ng/mL. LDT has been conducted after 3 weeks of treatment and 3 h after the gavage. Results are shown as mean ± SEM. Control (Water): *n* = 8; Safr’Inside: *n* = 9.

The behavioral phenotyping of mice chronically treated with Safr’Inside was extended to another symptomatic dimension of depression, namely anxious behavior as assessed in a standardized rodent anxiety test, the LDT [44]. As shown in Figure 5a,b, chronic Safr’Inside administration does not significantly alter the different anxiety-related parameters measured (i.e., latency to enter and time spent in the light box) (*p* > 0.1). Moreover, the relative distance covered during the test is similar in both groups, showing that Safr’Inside administration does not change locomotor activity (Figure 5c; *p* > 0.1). We also measured plasma level of corticosterone as a stress biomarker. Safr’Inside administration does not significantly alter corticosterone levels, which remain relatively low in the two groups (Figure 5d; *p* > 0.1). Taken together, these results show that in our experimental conditions Safr’Inside modulates depressive-like behavior, but not anxiety-like behavior or corticosterone levels.

### 3.3. Impact of Chronic Oral Administration of Safr’Inside^TM^ on Monoaminergic Neurotransmission

**Figure 6:**
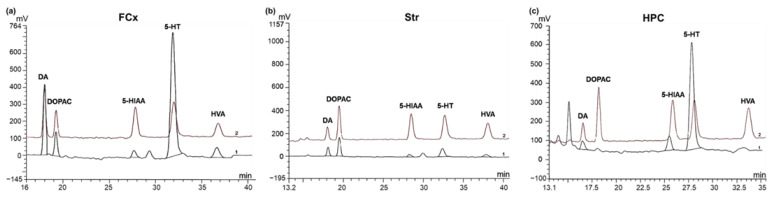
Representative chromatograms for the detection of dopamine (DA), 3,4-dihydroxyphenylacetic acid (DOPAC), homovanillic acid (HVA), serotonin (5-HT) and 5-hydroxyindolacetic acid (5-HIAA) by HPLC-EC. Signal overlay of one tested sample (1; black) and its corresponding standard solution (2; brown) in: (**a**) the Frontal Cortex (FCx); (**b**) the Striatum (Str) and; (**c**) the Hippocampus (HPC).

The next question was then to determine what neurobiological changes may underlie Safr’Inside-induced improvement of depressive-like behavior. For this purpose, we chose to focus on dopaminergic and serotonergic systems, whose dysfunction plays a pivotal role in the physiopathology of mood disorders [24,45]. We thus measured by HPLC-EC whole tissue levels of 5-HT, DA and their respective metabolites (5-HIAA; DOPAC and HVA) in three important brain areas for mood control: the FCx, Str and HPC (see representative chromatograms in Figure 6). The statistical analysis revealed that levels of monoamines and their metabolites differ according to the group (treated vs. controls) and the brain structure considered (Table 1). Overall, chronic Safr’Inside administration decreases levels of DA metabolites in the FCx, suggesting a reduction of DA degradation. Indeed, compared to the control group, mice treated with Safr’Inside display significantly lower levels of DOPAC (*p* ≤ 0.05) and HVA (*p* < 0.05). These results suggest that saffron may modulate DA neurotransmission in the FCx by reducing its catabolism. Interestingly, Safr’Inside administration also modulates DA neurotransmission in the Str, a major brain structure of the dopaminergic network, but this time by significantly increasing local DA levels (*p* ≤ 0.05), without any significant impact on its metabolites. In addition, no significant effect of chronic Safr’Inside gavage was found, neither on FCx, Str and HPC serotonergic systems, nor on HPC dopaminergic system. Indeed, DA levels were very low in the HPC as compared to the FCx and Str, while HPC DOPAC and HVA levels were undetectable whatever the group.

### 3.4. Impact of Chronic Oral Administration of Safr’Inside^TM^ on Gene Expression of Markers of 5-HT and DA Systems

**Figure 7:**
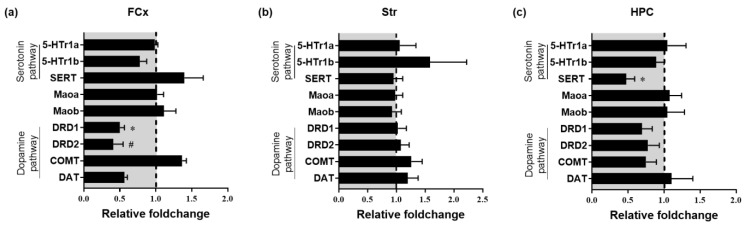
Effect of chronic oral administration of Safr’Inside^TM^ (6.25 mg/Kg) on gene expression of markers of serotonin and dopamine systems. Relative expression in: (**a**) the Frontal Cortex (FCx); (**b**) the Striatum (Str) and (**c**) the Hippocampus (HPC). Data are represented as the foldchange calculated relative to the control group (baseline = 1). Results are shown as mean ± SEM. Control (Water): *n* = 7–8; Safr’Inside: *n* = 8–9. # *p* = 0.05, * *p* < 0.05.

In order to study further the impact of Safr’Inside on 5-HT and DA neurotransmission, the expression levels of mRNAs coding for key regulatory factors of these systems, including different receptors (5-HTr1a, 5-HTr1b, DRD1 and DRD2), transporters (SERT and DAT) and degradation enzymes (Maoa, Maob and COMT), were measured in the same brain areas than HPLC analyses. In line with these last results, the impact of chronic Safr’Inside treatment on these markers differs depending on the brain area. No difference in expression levels was reported in the Str (Figure 7b), regardless of the gene. However, results confirmed that chronic Safr’Inside administration modulates FCx dopaminergic system (Figure 7a). Indeed, mRNA expression of DRD1 (*p* < 0.05) and DRD2 (*p* ≤ 0.05) is significantly decreased in the FCx of Safr’Inside-treated mice as compared to controls. Moreover, this treatment significantly decreases SERT expression in the HPC (Figure 7c; *p* < 0.05), which may decrease 5-HT reuptake. Taken together, these results show that chronic administration of Safr’Inside modulates brain dopaminergic and serotonergic neurotransmission, which may contribute to its concomitant beneficial effect on depressive-like behavior. 

## 4. Discussion

Although mounting clinical and preclinical studies pointed to the antidepressant-like properties of saffron [8,9,15,18,19,46], the underlying neurobiological mechanisms still remain largely unknown. The current study shows for the first time that oral administration of a saffron extract, Safr’Inside^TM^, improves depressive-like behavior and concomitantly modulates serotonergic and dopaminergic systems, known to contribute to the pathophysiology of mood disorders.

The FST is a well-validated behavioral paradigm routinely used for decades to assess the potential AD activity of candidate compounds in rodents [47]. It enables us to show here that a high dose of Safr’Inside (600 mg/Kg), which was selected based on previously published data [18], tends to improve depressive-like behavior as shown by reduced immobility index. However, it is worth mentioning that the clinical relevance of this result, as well as those obtained by other laboratories also using saffron extracts at high doses [18,19,46,48], is limited since they are hardly transposable to humans. Interestingly, it is not the case for the low dose (6.25 mg/Kg) tested in the present study, which was chosen based on the dose classically administered to humans (30 mg/day) using the guidelines for dose-equivalence calculation provided by the FDA [34]. To our knowledge, this is the first preclinical study reporting a positive effect on depressive-like behavior with such a low dose of saffron extract. This highlights further the translational relevance of our study.

In our second experiment, acute administration of the low dose of Safr’Inside displays a stronger behavioral effect in the FST than the chronic one. The experimental design applied, which led to expose mice twice to the same behavioral test can likely account for this unexpected result. This experimental choice was driven by the concern to replicate the results of the first experiment, while reducing the total number of mice used. It has been previously shown that exposing mice twice to the FST over a very short time period (a few days) decreases their specific behavioral response to the test [49]. Knowing that, we purposely used a much longer interval between the two FST sessions in our study (4 weeks), but the first test may still have influenced the second. More studies are needed to test this assumption by measuring the behavioral effect of chronic Safr’Inside administration in mice naïve to the FST. Meanwhile, the present study provides, beyond immobility, interesting information regarding active behaviors that can help to better understand the neurobiological mechanisms underlying the reported behavioral improvement. These active behaviors include swimming, which is particularly sensitive to serotonergic ADs such as selective 5-HT reuptake inhibitors (SSRIs), or climbing, which would be rather sensitive to noradrenergic drugs [50,51]. Here, Safr’Inside reduces immobility and concomitantly increases swimming time. Taken together, these results point to the serotonergic system as a likely player in the AD-like effect of Safr’Inside. Importantly, increased swimming time cannot be attributed to an unspecific psychostimulant property of Safr’Inside, since we show in the LDT that it does not alter locomotor activity. 

The behavioral responses displayed by Safr’Inside-treated mice in the FST could also be informative as to its mechanisms of action, in light of what is known about the behavioral impact of the different phytochemical compounds it contains, including crocins and safranal. Interestingly, it has been shown that crocins decrease the immobility time in the FST by especially increasing climbing time, while safranal increases both swimming and climbing [19]. The preferential action on swimming as compared to climbing reported in the present study therefore suggests that, among the different bioactive compounds of our extract, safranal could be particularly important to underlie the AD-like effect. This result does not totally discard however the potential contribution of crocins, the overall very short climbing time found in our study impeding any definitive conclusion to be drawn. This issue deserves to be studied in depth to decipher the respective role of each compound, while bearing in mind that, as previously hypothesized [19], the beneficial effects of saffron extracts on depressive-like behavior are likely mediated by the combined actions of their different bioactive constituents. 

While Safr‘Inside administration does improve depressive-like behavior, it does not reduce anxiety-like behavior, as assessed in chronically-treated mice exposed to a classical and pharmacologically validated rodent test of anxiety, the LDT [44]. It could be argued that a significant effect would have been seen after acute Safr‘Inside administration, as it happened regarding the FST. However, this is unlikely since we previously achieved a similar lack of anxiety improvement following acute treatment of another set of mice with the same dose of Safr‘Inside, although using another experimental paradigm (marble burying test) (see Supplementary Figure S1). In addition, it is noteworthy that controversial findings regarding the potential anxiolytic effects of saffron or its active compounds have been already reported in the literature [14,21,48]. The important experimental differences among these studies (e.g., different saffron extracts, doses and routes of administration, rats versus mice, distinct strains of mice with differential stress responsivity, etc.) prevent drawing a definitive conclusion on this topic, which still need to be thoroughly investigated. However, the present results clearly show that oral administration of Safr‘Inside at the dose of 6.25 mg/Kg does not improve anxiety-like behavior, at least when applied to unstressed mice that therefore display low baseline level of anxiety, as well as low corticosterone concentrations. Of note, those concentrations were similarly unchanged by our treatment in the present experimental conditions. Measuring circulating corticosterone is classically used as a stress biomarker related to anxiety. Interestingly, it has been previously reported that low doses of crocins, safranal, or saffron extract (1, 5 and 10 mg/Kg) prevent stress-induced increase in corticosterone levels and some of their related behavioral alterations [52,53], while a higher dose of crocins (30 mg/kg) is necessary to reduce basal corticosterone levels in naive rats [54]. These findings suggest that saffron may preferentially interfere with stress-induced activation of the HPA axis and related behavioral changes rather than with its basal activity. In line with this, the behavioral impact of our treatment might be stronger on stress-induced anxiety-like behavior rather than on basal anxiety. Supporting this assumption, we recently showed that chronic administration of Safr’Inside (6.25 mg/Kg) does reduce anxiety-like behavior in mice exposed to an unpredictable chronic mild stress protocol (see Supplementary Figure S2). 

One of the major strengths of this study was to combine the behavioral assessment with a detailed neurobiological characterization. As previously reported, depressive disorders have been associated with HPA axis dysregulation [55], which is targeted by most standard ADs [24]. The lack of effect of Safr’Inside on corticosterone levels reported here suggests however that it likely acts on other pathophysiological pathways to improve depressive-like behavior. Most conventional ADs aim to increase monoamine neurotransmission, primarily 5-HT, mainly by targeting their synaptic reuptake transporters or catabolic enzymes [56]. As mentioned above, behavioral changes displayed by Safr’Inside-treated mice in the FST point to modulation of serotonergic neurotransmission as an important player in the reported behavioral improvement. Supporting further this assumption, we showed that chronic Safr’Inside administration significantly reduces hippocampal expression of the SERT, which is consistent with an increase of 5-HT neurotransmission. This result is particularly interesting in light of several findings underlying the key role of hippocampal 5-HT in the therapeutic properties of SSRIs [57,58]. We did not demonstrate that changes in mRNA expression translated into changes in protein levels or activity, but previous studies have reported this to be the case, including for the SERT. For example, decreasing SERT mRNA expression with a siRNA treatment in mice was shown to induce a concomitant reduction of its binding sites, and to reproduce behavioral effects of SSRI in the FST [59]. Likewise, an in vitro study reports that an inflammatory treatment increased both the mRNA expression and reuptake activity of the SERT [60]. Here, reduced expression of SERT in the HPC is not associated with a detectable increase of local 5-HT concentrations. However, it is important to keep in mind that these measures were performed at whole tissue level, which means that 5-HT released in the synaptic cleft was not dissociated, at that stage, from the one stored in the presynaptic neurons. This assumption fits with recent findings reporting that alterations of SERT expression displayed by SERT^+/−^ rodents was not associated with detectable changes of basal 5-HT levels, but they did reduce SERT reuptake activity, confirming that changes in expression are well associated with functional modifications [61]. Although the present data need to be completed, particularly by performing dynamic microdialysis measures of monoamine levels, they already point to serotonergic neurotransmission as a likely target for AD-like effects of saffron. 

Importantly, we also provide key data suggesting that, together with 5-HT, saffron-induced modulation of dopaminergic neurotransmission could contribute to its behavioral effects, as reported for some standard ADs that also increase brain levels of DA [26]. An increase of brain DA levels following saffron administration has been previously reported [62], but this was only measured at the whole brain level and independently of any concomitant behavioral assessment. Here, we show for the first time that administration of Safr’Inside selectively increases DA levels in the Str, a major brain area of the mesolimbic dopaminergic pathway. In addition, it decreases DA metabolites levels (DOPAC and HVA) in the FCx, which is consistent with local reduction of DA degradation. Of note, no change in MAOs or COMT mRNA expression was observed, but this does not exclude a potential decrease in their enzymatic activity. Our treatment also lowers the mRNA expression of DRD1 and DRD2 in this same brain area, but we cannot determine at that time whether this is a direct effect of saffron or indirect consequences of the other dopaminergic changes it induced. It would also be nice to confirm these changes at the protein levels, although this has been already demonstrated in other experimental conditions [63,64]. In any case, all these results clearly indicate for the first time that chronic saffron administration is able to target different regulatory elements of the monoaminergic systems, depending on the monoamine and brain area considered, and suggest therefore that the resulting changes of 5-HT and DA neurotransmission likely contribute to improve depressive-like behavior.

Depressive disorders are increasingly viewed as a constellation of different symptoms domains (e.g., depressive, anxious, neurovegetative and somatic symptoms) with distinct underlying pathophysiological mechanisms [31]. In that context, the present study provides interesting information on the symptoms domains that may be preferentially improved by saffron. This especially concerns findings showing that Safr’Inside modulates monoaminergic systems, including part of the mesolimbic DA pathway that is involved in reward and motivation circuits [24]. Altered motivation, which is often reported in patients suffering from major depression [27], has been associated with dopaminergic alterations, including a decrease in striatal activity in response to reward [65]. Testing mice in the FST allowed revealing beneficial effects of saffron on depressive-like behavior related to despair or resignation. Nevertheless, it is not the most relevant approach to assess the potential effect of this treatment on motivation-related behaviors. Although this issue still need to be addressed, taken together, the present findings already suggest that saffron could also improve depressive symptoms related to impaired motivation. Interestingly, confirming this assumption may ultimately help to identify, on the basis of their clinical profile, depressed patients who could benefit from nutritional interventions with saffron. To go further, it would be particularly relevant to also check whether the different compounds isolated from saffron, which likely display distinct and/or complementary mechanisms of action [19], may exhibit differential efficacy depending on the symptoms domains. In the long run, this could enable to modulate the composition of saffron extract supplementations to finely fit with the clinical profile of depressed patients. Such potentially personalized nutritional strategies, which could be applied alone or as add-on treatment with SSRIs and/or dopaminergic ADs, could be notably valuable for the large proportion of patients that display poor response to pharmacological medications. These important issues deserve to be specifically addressed in future experiments by measuring the behavioral impact of the combination of saffron extracts and/or their different constituents with classical ADs. In support of this, administrating crocins together with SSRIs in patients with mild to moderate depression has been shown to improve the therapeutic effects of these ADs [16]. In addition, the present data, which highlight the combined action of our saffron extract on both dopaminergic and serotonergic systems, already suggest that it should likely modulate the therapeutic activity of classical ADs. Altogether, data from the present and future studies should open promising avenues for the development of new strategies to treat and manage depressive disorders. 

## 5. Conclusions

In conclusion, this study provides new and valuable findings on the beneficial effects of saffron on mood symptoms. Importantly, it allows to progress in the identification of the underlying neurobiological mechanisms by pointing to the involvement of serotonergic and dopaminergic systems. Further studies are needed to deeply understand how Safr’Inside can modulate the activity of these systems, as well as their causal role in the observed effects, and to test the contribution of other known pathophysiological bases of mood disorders. Nevertheless, we clearly report for the first-time that saffron-induced improvement of depressive-like behavior could be related to increased monoaminergic neurotransmission. Altogether, this work supports the therapeutic relevance of nutritional interventions with saffron extracts to improve the management and/or treatment of mood disorders. 

## Figures and Tables

**Table 1:** Effect of chronic oral administration of Safr’Inside^TM^ (6.25 mg/Kg) on monoamines levels measured by HPLC in the FCx, Str and HPC.

	FCx		Str		HPC	
(pmoles/g)	Control	Safr’Inside	Control	Safr’Inside	Control	Safr’Inside
[DA]	8988.1 ± 2875.0	4316.1 ± 1474.8	54,369.3 ± 8573.8	81,815.8 ± 9730.2 ^#^	244.8 ± 39.9	232.0 ± 60.2
[DOPAC]	1337.8 ± 141.9	914.1 ± 112.9 *	7237.3 ± 1295.3	9209.1 ± 1498.6	n.d	n.d
[HVA]	1915.0 ± 242.8	1287.4 ± 118.2 *	3135.9 ± 468.5	3979.2 ± 538.0	n.d	n.d
[5-HT]	1955.0 ± 324.3	1890.2 ±217.8	3536.3 ± 290.0	3282.2 ± 161.2	2101.3 ± 257.8	2069.6 ± 341.7
[5-HIAA]	464.1 ± 137.8	253.9 ± 29.5	749.7 ± 67.4	764.4 ± 62.0	442.5 ± 35.4	491.8 ± 103.8

The levels of dopamine (DA), 3,4-dihydroxyphenylacetic acid (DOPAC), homovanillic acid (HVA), serotonin (5-HT) and 5-hydroxyindolacetic acid (5-HIAA) are expressed in pmoles/g of tissue. Frontal Cortex (FCx), Striatum (Str) and Hippocampus (HPC). Results are shown as mean ± SEM. n.d: not detectable. Control (Water): *n* = 7–8; Safr’Inside: *n* = 7–9. ^#^ *p* = 0.05, * *p* < 0.05.

## Data Availability

The data presented in this study are available on request from the corresponding author.

## Supplementary Materials

The following information are available online at https://www.mdpi.com/2072-6643/13/3/904/s1, Supplementary Figure S1: Effect of acute oral administration of Safr’InsideTM (6.25 mg/Kg) on anxiety-like behavior measured in the marble burying test (MBT); Supplementary Figure S2: Effect of chronic oral administration of Safr’InsideTM (6.25 mg/Kg) on anxiety-like behavior measured in the marble burying test (MBT) on mice chronically exposed to Unpredictable Chronic Mild Stress (UCMS).
