# Supplementary material for: Saffron Extract-Induced Improvement of Depressive-Like Behavior in Mice Is Associated with Modulation of Monoaminergic Neurotransmission
Source: Nutrients. 2021 Mar 11;13(3):904. doi: 10.3390/nu13030904 (PMC8001199; doi:10.3390/nu13030904)
Supplement: Supplementary file 1 [file nutrients-13-00904-s001.pdf]

Supplementary Materials

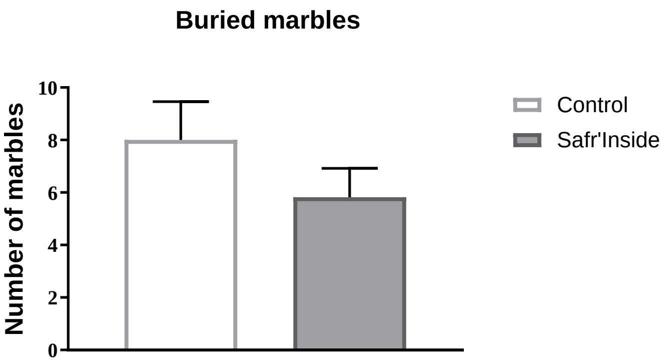

Supplemental Figure S1. *Effect of acute oral administration of Safr'Inside™ (6.25 mg/Kg) on anxiety-like behavior measured in the marble burying test (MBT).* MBT is a behavioral test used to assess anxiety-like behavior in rodents. It is based on the observation that they will spontaneously bury in their bedding objects perceived as potentially noxious, this behavior being reduced by anxiolytic drugs. Briefly, mice are placed for 30 min in a cage filled with 5 cm depth of bedding with 20 marbles evenly spaced. After 30 min, the number of marbles buried (2/3 of the marble covered) is counted. MBT has been conducted 30 min after gavage. Results are shown as mean ± SEM. Control (Water): n=10; Safr'Inside: n=11.

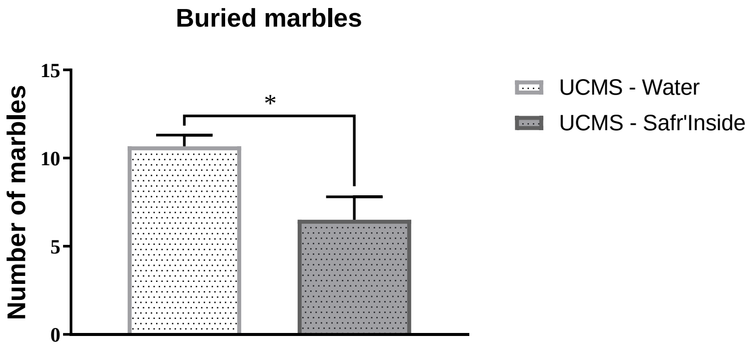

Supplemental Figure S2. *Effect of chronic oral administration of Safr'Inside™ (6.25 mg/Kg) on anxiety-like behavior measured in the marble burying test (MBT) on mice chronically exposed to Unpredictable Chronic Mild Stress (UCMS) for 7 weeks.* Number of buried marbles out of the 20 total marbles after 30-min test. Safr'Inside treatment began at the 4th week of stress. MBT was conducted after 3 weeks of daily treatment and 3 hours after gavage. Results are shown as mean ± SEM. UCMS - Water: n=9; UCMS - Safr'Inside: n=11. *$p < 0.05$
